# Outcomes of adolescents and young adults with chronic-phase chronic myeloid leukaemia treated with tyrosine kinase inhibitors

**DOI:** 10.1080/07853890.2022.2069280

**Published:** 2022-04-29

**Authors:** Yuriko Nishiyama-Fujita, Tomonori Nakazato, Noriyoshi Iriyama, Michihide Tokuhira, Maho Ishikawa, Eriko Sato, Tomoiku Takaku, Keiji Sugimoto, Hiroyuki Fujita, Isao Fujioka, Shun Tsuchiya, Yuta Kimura, Eisaku Iwanaga, Norio Komatsu, Norio Asou, Masahiro Kizaki, Yoshihiro Hatta, Tatsuya Kawaguchi

**Affiliations:** a Department of Hematology, Yokohama Municipal Citizen’s Hospital, Yokohama, Japan; b Department of Medicine, Division of Hematology and Rheumatology, Nihon University School of Medicine, Tokyo, Japan; c Department of Hematology, Saitama Medical Center, Saitama Medical University, Saitama, Japan; d Department of Hemato-Oncology, International Medical Center, Saitama Medical University, Saitama, Japan; e Department of Hematology, Juntendo University Nerima Hospital, Tokyo, Japan; f Department of Hematology, Juntendo University School of Medicine, Tokyo, Japan; g Department of Hematology, Juntendo University Urayasu Hospital, Urayasu, Japan; h Department of Hematology, Saiseikai Yokohama Nanbu Hospital, Yokohama, Japan; i Department of Hematology, Kumamoto University Hospital, Kumamoto, Japan; j Medical Technology, Kumamoto Health Science University, Kumamoto, Japan

**Keywords:** Chronic phase chronic myeloid leukaemia, adolescents and young adults, tyrosine kinase inhibitors, Dasatinib, Nilotinib

## Abstract

**Introduction:**

Few studies have reported the outcomes of adolescents and young adults (AYAs) with chronic-phase chronic myeloid leukaemia (CML-CP) on tyrosine kinase inhibitors (TKIs).

**Materials and methods:**

We retrospectively analysed the clinical features, treatment response, and long-term outcomes of 42 AYA patients, in comparison to older patients. The initial therapies of AYA patients between 2001 and 2016 included imatinib (*n* = 24), dasatinib (*n* = 13) and nilotinib (*n* = 5).

**Results:**

In AYA patients, the peripheral blood (PB) white blood cell count and percentage of blasts at the diagnosis were significantly higher, haemoglobin levels were lower and the spleen size was larger. The major molecular response (MMR), event-free survival (EFS) and overall survival (OS) rates were comparable. A sub-analysis comparing imatinib to second-generation TKIs as the initial therapy also showed that their prognosis was comparable.

**Discussion:**

In conclusion, the tumour burden at the diagnosis of CML-CP is higher in AYA patients; however, their prognosis was not worse in comparison to older patients treated with TKIs.

**KEY MESSAGES**
Few studies have reported the outcomes of adolescents and young adults (AYAs) with chronic-phase chronic myeloid leukaemia (CML-CP) on tyrosine kinase inhibitors (TKIs). This study showed the tumour burden at the diagnosis of CML-CP is higher in AYA pa tients; however, their prognosis was not worse in comparison to older patients treated with TKIs. Understanding the biological and non-biological features of AYA patients with CML-CP on TKI therapy is essential for better management and to improve the outcomes.

## Introduction

The outcomes of chronic myeloid leukaemia (CML) have dramatically improved since the introduction of tyrosine kinase inhibitor (TKI) treatment. Although the median age of chronic-phase CML (CML-CP) patients is reported to be 62 years [1], there is a small but significant number of younger patients, including a group of patients referred to as adolescents and young adults (AYAs), which is defined as 15–39 years of age [2–4]. Although cancer is predominantly a disease of older adults and the elderly, AYAs represent approximately 40% of the world population and it is estimated that there are 1 million new cancer diagnoses in AYAs each year [5,6].

Many AYA patients continue to experience inferior outcomes in comparison to younger and older age groups for many reasons. One of the reasons is reported to be low clinical trial participation resulting in a low rate of tumour specimen acquisition for research [7,8]; thus, there is less understanding of the biological features in this patient population. Other reasons include fewer opportunities to receive medical check-ups, lack of standardized therapeutic approaches, poor compliance with therapy, psychosocial issues, including fertility preservation, contraception before the initiation of therapy, increased risk of mental health disorders, quality-of-life (QOL) issues, insurance or financial issues and the availability and identification of resources [9–11]. CML is no exception in this regard, and little is known about the outcomes of AYA patients with CML, particularly in the TKI era.

To our knowledge, few studies have precisely analysed the outcomes of AYA patients with CML-CP treated with TKIs, including the second-generation TKIs nilotinib and dasatinib **[**12–17**]**. We therefore retrospectively analysed the clinical characteristics and outcomes of AYA patients with CML-CP treated with TKIs and further evaluated the outcomes of AYA patients who were treated with nilotinib, and dasatinib.

## Materials and methods

### Patients

A retrospective review of data from the CML Cooperative Study Group, which includes four University Hospitals and four University Branch Hospitals, was performed. Our study included 360 patients of ≥ 18 years of age who were diagnosed with CML-CP between April 2001 and January 2016 and who were treated with any TKIs. CML-CP was diagnosed according to the European Leukaemia Net (ELN) criteria, as described previously [18]. Patients in the accelerated phase (AP) or blast phase (BP) were excluded from this study. As the majority of the reported studies have used 29 years of age as a cut-off value for AYAs in cohort analyses, we used a cut-off value of 29 years to improve the integrity of the scientific questions, including the patient characteristics, treatment response and long-term outcomes. The response criteria were as previously described [19]. An MMR was defined as a *MAJOR BCR-ABL1* mRNA*/ABL1* mRNA ratio of ≤0.1%, as determined by the International Scale (IS), and a DMR was defined as a *MAJOR BCR-ABL1* mRNA/*ABL1* mRNA ratio of ≤0.0032%, as determined by the IS. We could not collect details about the achievement of a complete cytogenetic response (CCyR) for all patients in this study. The study was approved by the ethics committees of all hospitals involved and was conducted in accordance with the Declaration of Helsinki. Written informed consent was obtained from all the patients after fully explaining the nature of the study and that the information of the patients should not be identified. The grade of each side effect was based on the Common Terminology Criteria for Adverse Events (CTCAE) version 4.0 (National Cancer Institute, Maryland, USA).

### Statistical analyses

Differences among variables were evaluated by the *χ*^2^ test and Mann–Whitney U test for categorical and continuous variables, respectively. OS was defined as the period between the date of the initial TKI treatment and the date of death due to any cause. EFS was measured from the start of the initial treatment to the date of any of the following events while on therapy: loss of a complete haematologic response, loss of a complete or major cytogenetic response, or progression to AP or BP, and death from any cause at any time. The probability of survival was estimated by the Kaplan–Meier method and compared by the log-rank test. The MMR and DMR were assessed, regardless of whether treatment agents were switched, and were evaluated as the probability of attaining an MMR or DMR at each time point. The comparison between the cumulative incidence of an MMR or DMR, or transformation to AP or BP was performed using the Grey test [20]. *P* values of < .05 were considered to indicate statistical significance. All statistical analyses were performed using the EZR software program [21].

## Results

### Patient characteristics

Data from 360 patients (male, *n* = 221 [61%]; female, *n* = 139 [39%]) were analysed. The median follow-up period was 67 months (range, 0–202 months). The median age at the time of the diagnosis of CML was 53.5 years (range, 18–86 years). A total of 182 patients were treated with imatinib, 80 were treated with nilotinib and 98 were treated with dasatinib as the initial treatment. The 360 patients were divided into two groups according to their age at the time of the diagnosis. Patients who were diagnosed with CML-CP at 18–29 years of age were classified into the AYA group (*n* = 42; 11.7%) and those who were diagnosed at ≥ 30 years of age were classified into the older group (*n* = 318; 88.3%). The demographics and baseline characteristics of the two patient groups are shown in Table 1. The AYA group showed a significantly larger spleen size (*p* < .0001), higher white blood cell count (*p* < .0001), a higher percentage of lymphocytes (*p* = .008), a higher percentage of blasts in peripheral blood (PB) (*p* < .0001), and a lower haemoglobin level (*p* = .004). They also showed higher probability of having a high-risk status according to the Sokal and Hasford scores (*p* = .009 and .001, respectively.) There was no difference in the percentages of eosinophils or basophils in the PB, the platelet count or the European Treatment and Outcomes Study (EUTOS) or EUTOS long-term survival (ELTS) scores at the time of the diagnosis. The proportions of patients who received each TKI were comparable in the AYA and older groups. Twenty-four (57.1%), 13 (31.0%) and 5 (11.9%) among total of 42 AYA group patients and 158 (49.7%), 85 (26.7%) and 75 (23.6%) among total of 318 older group patients were treated with imatinib, dasatinib and nilotinib, respectively, as the initial treatment. The median observation period was 76 months (range: 0–173 months) in the AYA group and 66 months (range: 4–202 months) in the older group (*p* = .676). The older group included one patient with platelet depletion (<100 × 10^9^/L) due to liver cirrhosis, but not disease progression. In some cases, the Sokal, EUTOS, Hasford and ELTS score data were missing

**Table 1.** Patient characteristics according to the age at the time of treatment initiation.

Factor	Age 18–29 (AYA) (*N* = 42)	Age 30–89 (*N* = 318)	*p* Value
Median age	25	57	.0001
Sex (male/female)	26/16	195/123	1
Spleen size below costal margin (cm)	5.65 ± 7.61	1.16 ± 3.53	<.0001
WBC (×10^9^/L)	102.1 (10.8–719.8)	31.8 (5.0–482.4)	<.0001
Haemoglobin (g/dL)	11.4 (5.8–16.1)	13.1 (5.0–18.8)	.004
Platelet (×10^9^/L)	489 (118–2282)	506 (86–4352)	.758
PB (%)			
Eosinophils	2.0 (0–10)	2.0 (0–24)	.765
Basophils	4.6 (0–17)	5.5 (0–19.5)	.06
Blasts	0.5 (0–13.0)	0 (0–13.5)	<.0001
Lymphocytes	4.3 (0–22.0)	8.5 (0–37.3)	.008
Sokal score, *n* (%)			
Low	21 (50%)	127 (40%)	.009
Intermediate	5 (12%)	123 (39%)	
High	13 (31%)	49 (15%)	
NE	3 (7%)	19 (6%)	
EUTOS score, *n* (%)			
Low	30 (72%)	263 (83%)	.141
High	9 (21%)	40 (12%)	
NE	3 (7%)	15 (5%)	
ELTS score, *n* (%)			
Low	27 (64.3)	223 (70.1)	.307
Intermediate	7 (16.7)	66 (20.8)	
High	4 (9.5)	14(4.4)	
NE	4 (9.5)	15 (4.7)	
Hasford score, *n* (%)			
Low	23 (55%)	120 (38%)	.001
Intermediate	9 (21%)	158 (50%)	
High	7 (17%)	25 (8%)	
NE	3 (7%)	15 (5%)	
Agent, *n* (%)			
Imatinib	24 (57.1%)	158 (49.7)	.231
Dasatinib	13 (31.0)	85 (26.7)	
Nilotinib	5 (11.9)	75 (23.6)	
Observation period, median (month)	76 (0–173)	66 (4–202)	.676

AYA: adolescents and young adults; PB: peripheral blood; EUTOS: European Treatment and Outcome Study; ELTS: EUTOS long-term survival; NE: not evaluated

### Treatment response

**Figure 1.**
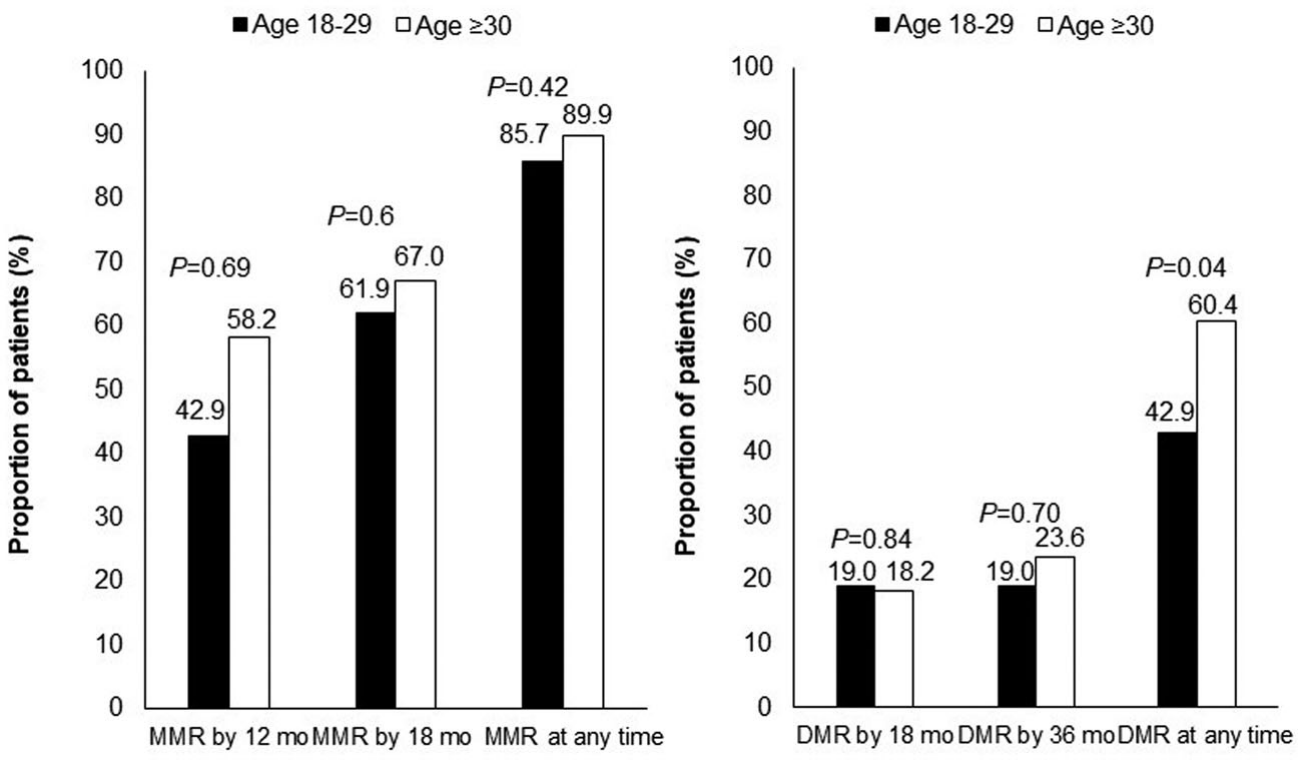
Molecular responses to TKIs according to age group. *p* Refers to the level of significance between the AYA and older groups. MMR: major molecular response; DMR: deep molecular response; TKI: tyrosine kinase inhibitor.

**Figure 2.**
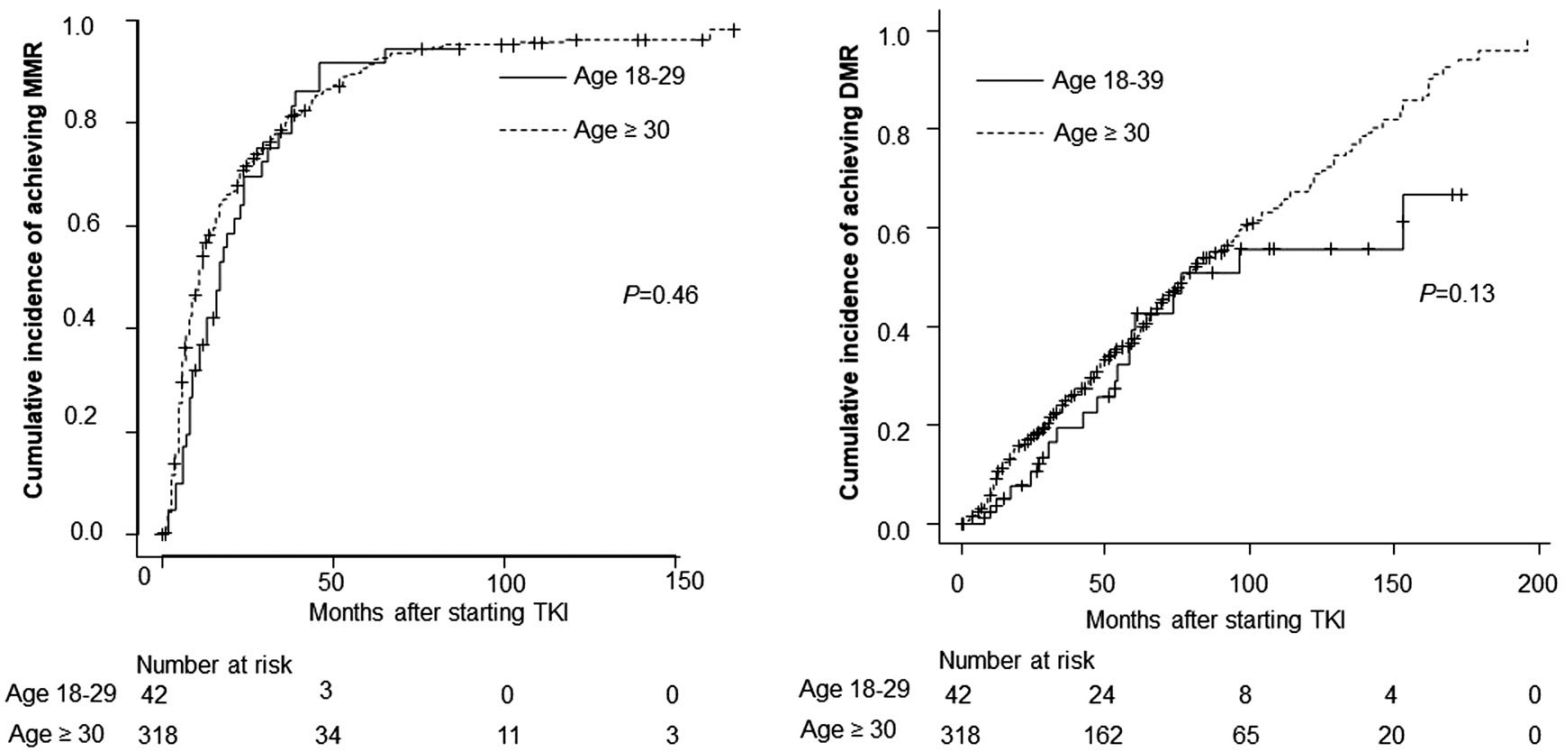
Response to TKI according to age group. (A) Cumulative incidence of MMR. (B) Cumulative incidence of DMR. *p* Refers to the level of significance between the AYA and older groups. MMR: major molecular response; DMR: deep molecular response; TKI: tyrosine kinase inhibitor.

The treatment responses of the AYA and older groups were comparable. The MMR rate at all timepoints during the follow-up period was 85.7% in the AYA group and 89.9% in the older group (*p* = .42). The DMR rate at all timepoints during the follow-up period was 42.9% in the AYA group and 60.4% in the older group (*p* = .04). The MMR rates at 12 months and 18 months did not differ between the AYA and older groups (MMR at 12 months: 42.9 and 58.2%, respectively, *p* = .69. MMR at 18 months: 61.9 and 67.0%, respectively, *p* = .6) (Figure 1). The cumulative incidence of MMR and DMR was comparable between the 2 groups (Figure 2).

### Long-term outcomes

**Figure 3.**
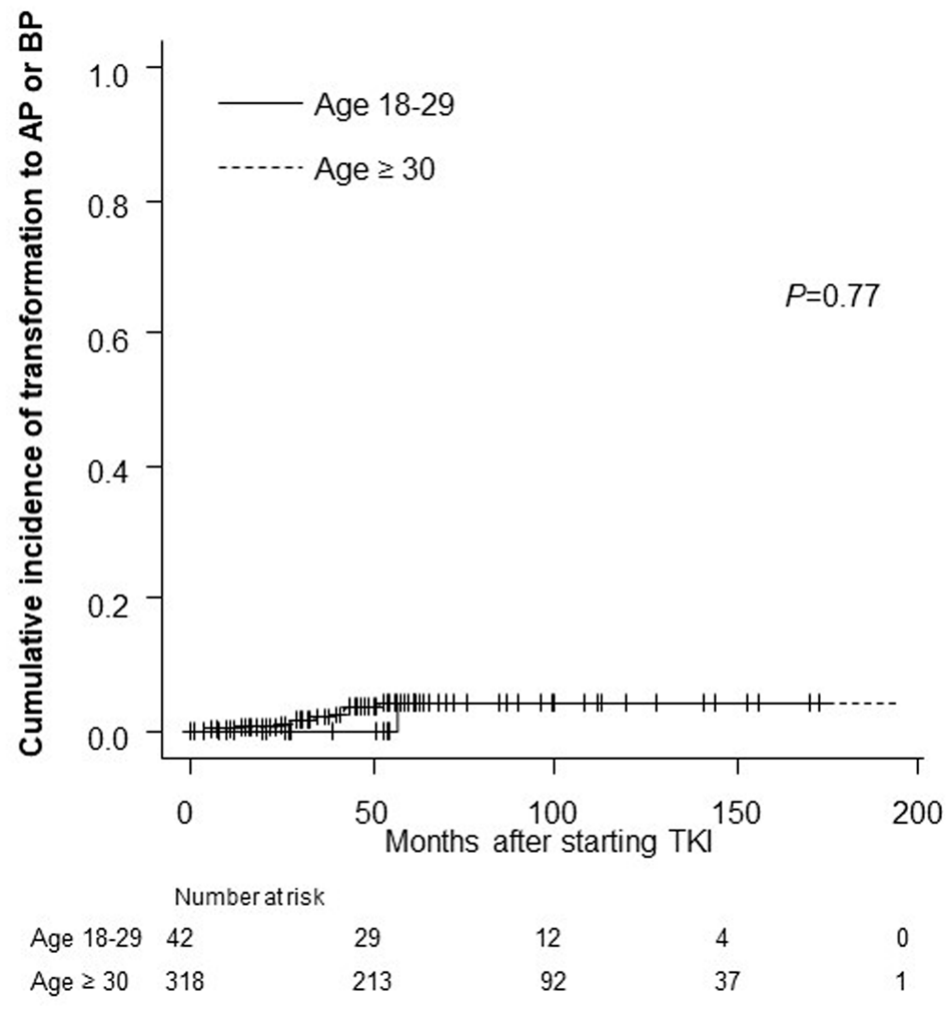
Cumulative incidence of transformation to AP or BP according to age group. *p* Refers to the level of significance between the AYA and older groups. AYA: adolescents and young adults; AP: accelerated phase; BP: blast phase; TKI: tyrosine kinase inhibitor.

**Figure 4.**
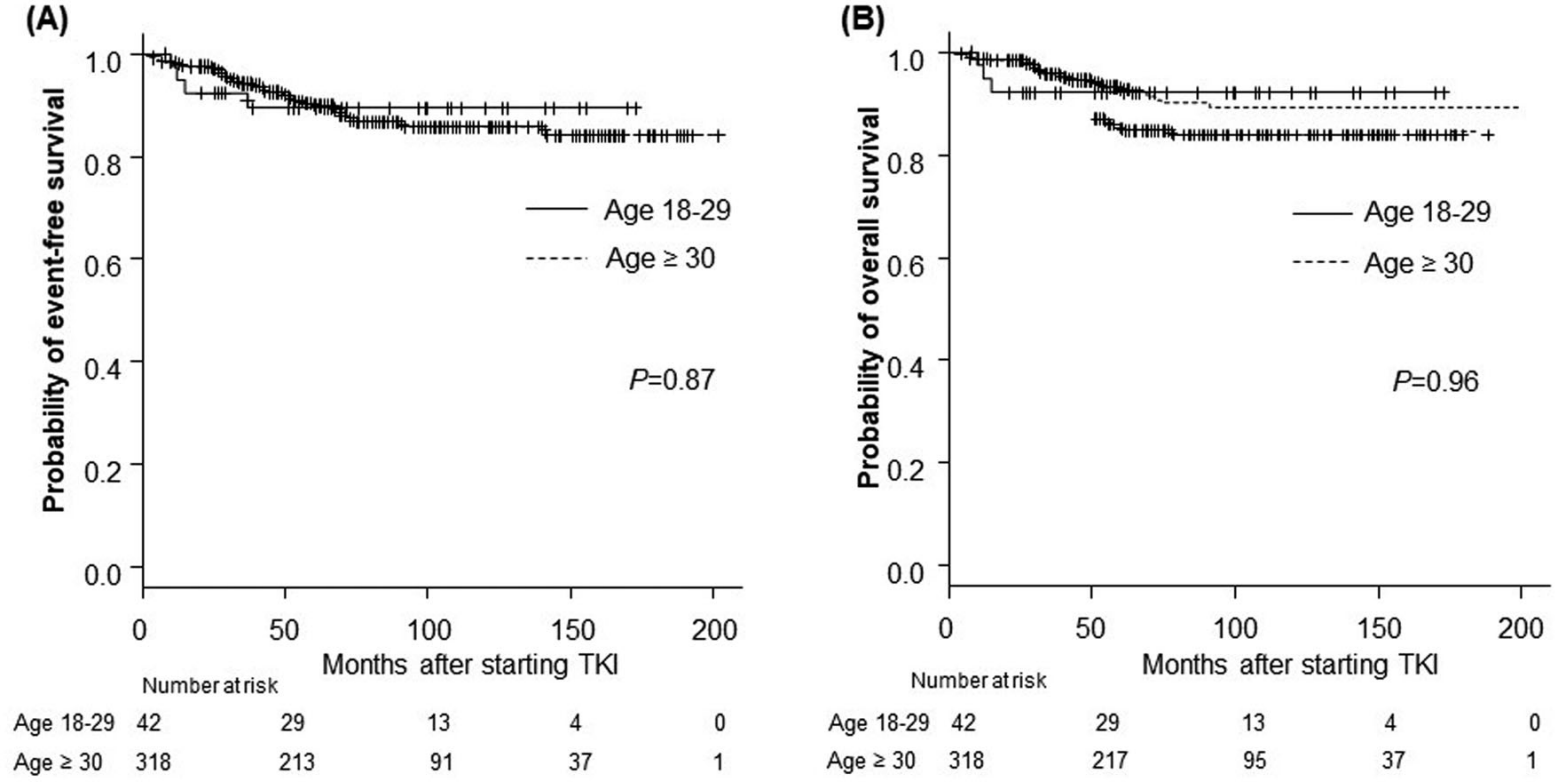
Long-term outcomes according to age group among patients treated with TKIs. (A) The 5-year event-free survival (EFS) rate of the AYA group: 89.3%; older group: 89.8% (*p* = .87). (B) The 5-year overall survival (OS) rate of the AYA group: 92.3%; the older group: 92.8% (*p* = .96). *p* Refers to the level of significance between the AYA and older groups. AYA: adolescents and young adults.

The cumulative incidence of transformation to AP or BP in the AYA and older groups did not differ to a statistically significant extent (*p* = .77, Figure 3). The EFS and OS rates were also comparable between the AYA and older groups. The 5-year EFS rate was 89.3% in the AYA group and 89.8% in the older group (*p* = .87) (Figure 4(A)). The 5-year OS rate was 92.3% in the AYA group, and 92.8% in the older group (*p* = .96) (Figure 4(B)). The rates of progression and leukaemia-related or leukaemia-unrelated death are shown in Table 2. The rates of leukaemia-unrelated death in the AYA and older groups were 4.8% and 4.7%, respectively (Table 2 near here).

**Table 2.** The rates of progression and leukaemia-related or leukaemia-unrelated death according to age group.

	Total	Progression to AP or BP		Leukaemia-related death		Leukaemia-unrelated death		Total death	
Age group	*N*	*N*	(%)	*N*	(%)	*N*	(%)	*N*	(%)
Age 18–29 (AYA)	42	1	(2.4)	1	(2.4)	2	(4.8)	3	(7.1)
Age ≥ 30	318	11	(3.5)	9	(2.8)	15	(4.7)	24	(7.5)
Total	360	12	(3.3)	10	(2.8)	17	(4.7)	27	(7.5)

Progression to AP or BP is defined by European Leukaemia Net (ELN) criteria. All leukaemia-related death occurred after transformation to AP or BP.

AYA: adolescents and young adults; AP: accelerated phase; BP: blast phase

### The incidence and reasons for dose reduction or switching of initial TKIs

We investigated the incidence and reasons for dose reduction or switching of the initial TKIs. The rate of dose reduction in the AYA group was lower than that in the older group (4.7 *vs.* 39.6%). The reasons for intolerance in the AYA group were skin rash and oedema with imatinib. For the older group, the common reasons for intolerance were skin rash and patient request for imatinib, and pleural effusion with dasatinib (Supplementary Table 1).

After a median follow-up period of 67 months from the start of the initial treatment, 273 patients (75.8%) continued to receive their initial TKI. TKIs were more frequently switched in the AYA group than in the older group (57.1 *vs.* 19.8%) (Supplementary Table 2). Sixteen of the 24 imatinib-treated patients in the AYA group (66.7%) switched to a second-generation TKI, including nilotinib (*n* = 7) and dasatinib (*n* = 8) or IFN-α due to pregnancy (*n* = 1). Seven of the 13 dasatinib-treated patients in the AYA group (76.9%) switched to other TKIs, including imatinib (*n* = 1), nilotinib (*n* = 3) or bosutinib (*n* = 1), or registering in the STOP TKI study (*n* = 2). One of the five nilotinib-treated patients in the AYA group (20%) switched to dasatinib. The reasons of switching from imatinib were resistance (*n* = 5), the achievement of a DMR (*n* = 5), registering in the STOP-TKI study (*n* = 1), and intolerance due to unknown reasons (*n* = 4). The reasons for switching from dasatinib or nilotinib were similar to those of imatinib.

Thirty-seven of the 158 imatinib-treated patients in the older group (23.4%) switched from imatinib to second-generation TKIs including nilotinib (*n* = 19) and dasatinib (*n* = 16), or third-generation TKIs bosutinib (*n* = 2). Seventeen of the 85 dasatinib-treated patients in the older group (20%) switched to other TKIs, including imatinib (*n* = 2), nilotinib (*n* = 9) or bosutinib (*n* = 6). Nine of the 75 nilotinib-treated patients in the older group (12%) switched to dasatinib (*n* = 7) or imatinib (*n* = 2). The reasons of switching from imatinib were resistance (*n* = 7), the achievement of a DMR (*n* = 5), muscle pain (*n* = 1), renal dysfunction (*n* = 2), pleural effusion (*n* = 1), oedema (*n* = 2), nausea (*n* = 1), skin rash (*n* = 1), diarrhoea (*n* = 1), patient’s request (*n* = 1), registering in the STOP-TKI study (*n* = 2) and intolerance due to unknown reasons (*n* = 13). The reasons for switching from dasatinib were resistance (*n* = 1), the achievement of a DMR (*n* = 1), hepatic dysfunction (*n* = 1), drug-induced lung injury (*n* = 1), renal dysfunction (*n* = 1), pleural effusion (*n* = 4), skin rash (*n* = 1), cytopenia (*n* = 1), drug-induced colitis (*n* = 1) and intolerance due to unknown reasons (*n* = 5). The reasons for switching from nilotinib were resistance (*n* = 2), oedema (*n* = 1), IgG4-related sclerosing cholangitis (*n* = 1), diabetes mellitus (*n* = 1), heart failure (*n* = 1), creatinine kinase elevation (*n* = 1), chest pain (*n* = 1) and intolerance due to unknown reasons (*n* = 1).

### Sub-analysis according to the TKI used for the initial therapy

#### Treatment response

**Figure 5.**
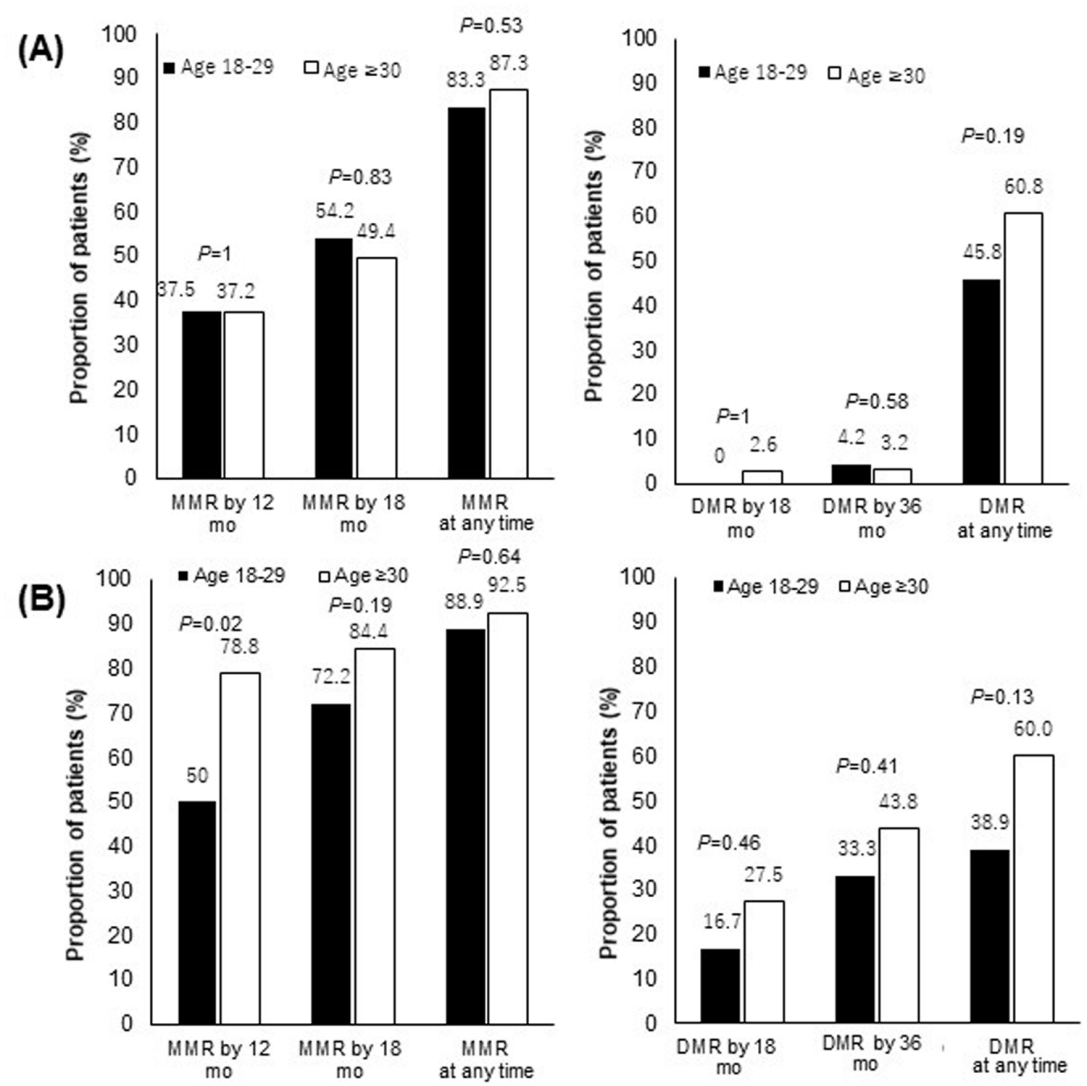
Molecular responses to each TKI according to age group. (A) Molecular responses according to age group among patients treated with imatinib as an initial therapy (*N* = 182). (B) Molecular responses according to age group among patients treated with nilotinib or dasatinib as initial therapy (*N* = 178). *p* Refers to the level of significance between the AYA and older groups. AYA: adolescents and young adults; MMR: major molecular response; DMR: deep molecular response.

Among patients who received imatinib as the initial therapy, no significant difference was seen in the MMR or DMR rates between the AYA group and older groups. The MMR rate at all timepoints during the follow-up period was 83.3% in the AYA group and 87.3% in the older group (*p* = .53). The DMR rate at all timepoints during the follow-up period was 45.8% in the AYA group and 60.8% in the older group (*p* = .19) (Figure 5(A)).

Furthermore, among patients treated with dasatinib or nilotinib as the initial therapy, no significant difference was seen in the MMR or DMR rates of the AYA and older group. The MMR rate at all timepoints during the follow-up period was 88.9% in the AYA group and 92.5% in the older group (*p* = .64). The DMR rate at all timepoints during the follow-up period was 38.9% in the AYA group and 60.0% in the older group (*p* = .13) (Figure 5(B)).

#### Long-term outcomes

**Figure 6.**
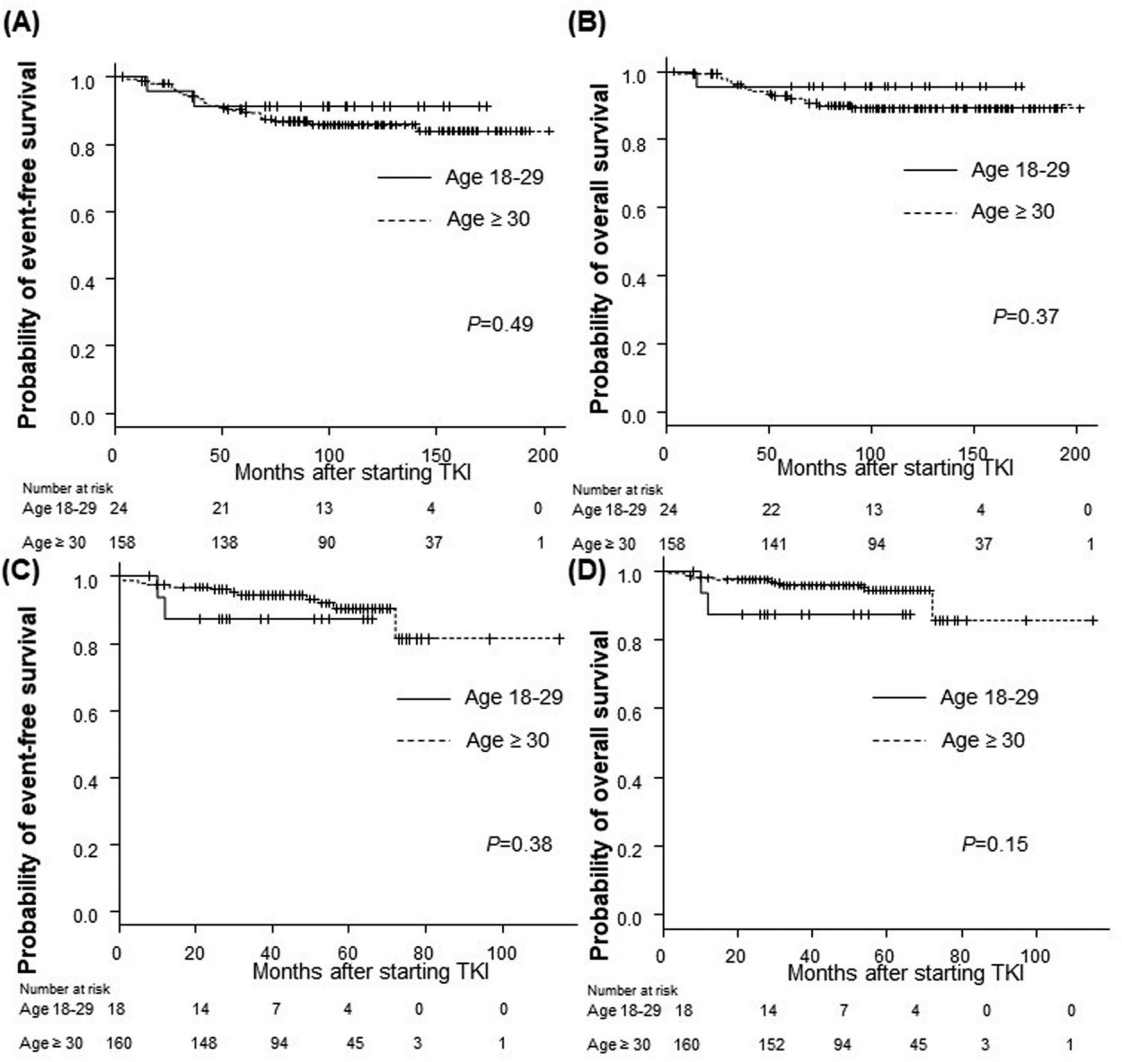
Long-term outcomes according to age group among patients treated with each TKI. (A,B) Imatinib as initial therapy, (C,D) nilotinib or dasatinib as initial therapy. (A) The 5-year event-free survival (EFS) rate of the AYA group: 91.3%; the older group: 89.5% (*p* = .49). (B) The 5-year overall survival (OS) rate of the AYA group: 95.7%; the older group: 92.0% (*p* = .37). (C) The 5-year EFS rate of the AYA group: 87.5%; the older group: 90.3% (*p* = .38). (D) The 5-year OS rate of the AYA group: 87.5%; the older group: 94.4% (*p* = .15). *p* Refers to the level of significance between the AYA and older groups. AYA: adolescents and young adults; TKI: tyrosine kinase inhibitor.

The EFS and OS rates of the AYA and older groups were comparable in patients who received imatinib and those who received second-generation TKIs as the initial therapy. Among the patients who were treated with imatinib as the initial therapy, the 5-year EFS rate was 91.3% in the AYA group and 89.5% in the older group (*p* = .49), the 5-year OS rate was 95.7% in the AYA group, and 92.0% in the older group (*p* = .37) (Figure 6(A,B)). Among patients treated with dasatinib or nilotinib as the initial therapy, the 5-year EFS rate was 87.5% in the AYA group and 90.3% in the older group (*p* = .38), the 5-year OS rate was 87.5% in the AYA group and 94.4% in the older group (*p* = .15) (Figure 6(C,D)).

## Discussion

This study showed that the tumour burden at the diagnosis of CML-CP was higher in the AYA group, but that the prognosis was not worse in comparison to the older group.

There has been no definite conclusion on the clinical appearance or the outcome of AYA patients with CML-CP treated with TKIs, including second-generation TKIs. Several studies have examined the clinical characteristics and outcomes of AYA patients with CML-CP (Table 3). Kalmanti et al. assessed a total of 1524 CML-CP patients treated with imatinib or imatinib and interferon (IFN)-α, and reported that AYA patients showed comparable MMR and DMR rates and OS [12]. On the other hand, several studies have reported a lower treatment response in AYA patients. Latagliata et al. assessed 206 CML-CP patients treated with imatinib and reported inferior MMR rates and shorter EFS in AYA patients [13]. In a cohort of the GIMEMA study, which analysed 774 patients treated with imatinib or the second generation TKI, nilotinib, the MMR rate of AYA patients was lower than that of older patients [14]. Both studies showed no significant difference in OS. In contrast, other studies have demonstrated not only a lower treatment response but also inferior long-term outcomes in AYA patients with CML-CP (Table 3 near here).

**Table 3.** Studies on the clinical characteristics and outcomes of AYA patients with CML-CP.

Institute country	Initial treatment (*N*)	Age group	(*N*)	MMR	DMR	EFS (%)	OS (%)	Author reference
CML-CSG Japan	Imatinib (182) Nilotinib (80) Dasatinib (98)	Total 18–29 y ≥30 y	360 42 318	85.7% 89.9% (*p* = .42)	42.9% 60.4% (*p* = .04)	(5 y) 89.3 89.8 (*p* = .87)	(5 y) 92.3 92.8 (*p* = .96)	Our study
Germany	Imatinib or Imatinib +IFN-α	Total 16–29 y 30–44 y 45–59 y ≥60 y	1524 120 383 495 526	(Median) 17.6 mo 17.4 mo 15.7 mo 16.5 mo	(Median) 39.0 mo 36.8 mo 33.0 mo 38.7 mo		(5 y) 96.7 93.8 92.5 82.9 (*p* < .001)	[12]
Italy	Imatinib	Total 20–44 y 45–64 y ≥ 65 y	206 61 72 73	58.4% 86.5% 57.4% (*p* < .001)		(4 y) 67.3 92.0 61.1 (*p* = .001)	(4 y) 96.3 100 72.4 (*p* < .001)	[13]
GIMEMA Italy	Imatinib Nilotinib	Total 18–29 y 30–59 y ≥ 60 y	774 56 457 261	71% 86% 88% (*p* = .004)			(8 y) 93% 93% 77% (*p* < .001)	[14]
USA	Imatinib (281) Nilotiib (98) Dasatinib (89)	Total 15–29 y ≥ 30 y	468 61 407	75% 86% (*p* = .049)	23% 41% (*p* = .01)	(5 y) 71 82 (*p* = .07)	(5 y) 95 93 (*p* = .35)	[15]
Japan	Imatinib (120) Nilotinib (7) Dasatinib (6)	Total 15–29 y ≥30 y	133 19 114	60.5% 87% (*p* < .05)	17.4% 33.4% (*p* < .05)	(7 y) 58.1 80.1 (*p* = .02)		[16]

CML-CP: chronic-phase chronic myeloid leukaemia; AYA: adolescents and young adults; MMR: major molecular response; DMR: deep molecular response; EFS: event-free survival; OS: overall survival; y: years of age; mo: months; IFN-α: interferon-alpha; GIMEMA: Gruppo Italiano Malattle Ematologiche d’Adulto

Definition of DMR is variable in each study, and it is set as MR4.0 or MR4.5 or undetectable by in-house qualitative reverse transcription-polymerase chain reaction.

The study by Kalmanti et al. presented the MMR and DMR as a time to the achievement of median cumulative incidence of MMR and DMR, respectively.

In these previous studies, most patients were treated with imatinib, and patients treated with a second-generation TKI as an initial treatment only accounted for a small percentage of the patients in each study. However, the ENEST1st study, which included 1091 CML-CP patients in an open-label, multicentre single-arm, prospective study on nilotinib, indicated that age did not have a relevant impact on the DMR rate [17].

We analysed AYA patients with CML-CP who were treated with imatinib or second-generation TKIs (nilotinib or dasatinib). In contrast to the previous studies, with the exception of the ENEST1st study, the MMR and EFS rates of the AYA group were not inferior to those of the older group. One of the possible reasons of this is that the rate of TKI switching was higher in the AYA group; 66.7 *vs.* 23.4% of imatinib-treated patients in the AYA group and the older group respectively (Supplementary Table 2). More treatment options were available during this study with the availability of second-generation TKIs. Seventy percent of the patients were treated after March 2009, when second-generation TKIs were approved in Japan. Other than resistance and intolerance, one of the reasons of switching from imatinib was the achievement of DMR. Achieving a deeper response faster has been associated with improved outcome in general. Inducing durable DMR may potentially lead to therapy discontinuation which is especially important for AYA patients, and second-generation TKIs induce higher rate of DMR [22,23]. According to the German CML-IV study, patients with a confirmed DMR by 4 years had a higher rate of 8-year OS in comparison to patients with CCyR without an MMR by 4 years, and no patients who achieved a DMR experienced disease progression [23]. In another study, patients who achieved a DMR had higher rates of EFS and failure-free survival than patients who achieved a CCyR without a DMR [24].

Although it was not statistically significant, the factor that was associated with the inferior trend of DMR rate in the AYA group seems to be their higher tumour burden at the time of the diagnosis. The AYA group had a high rate of spleen enlargement and higher percentages of blasts in their PB in comparison to the older group, which confirmed the findings reported in previous studies [12,14,15,17]. In this study, the AYA group also had a significantly higher median WBC count, which was in line with the report by Latagliata et al. [13]. A possible explanation for the higher tumour burden of the AYA group at the diagnosis is the delayed diagnosis of this particular group in comparison to the older group who undergo routine check-ups, including blood-test, and who have more opportunities to undergo medical examinations as they often have comorbidities. Among the AYA group, these features were more prominent in patients who could not reach a DMR in comparison to those who could reach a DMR. At the time of the diagnosis, AYA patients who could not reach a DMR showed an enlarged spleen size (length below costal margin 8.6 *vs.* 0.8 cm, *p* = .0005), higher WBC count (217.2 × 10^9^ *vs*. 59.9 × 10^9^ cells/L, *p* = .0005), lower haemoglobin level (11.0 *vs.* 13.1 g/dL, *p* = .008), and a lower percentage of lymphocytes in their PB (5.0 *vs.* 9.5%, *p* = .04) in comparison to those who could reach a DMR. These features may have prognostic value for AYA patients with CML-CP. Although the cumulative incidence of a DMR in the two groups was comparable, the cumulative incidence in the AYA group slowed down at around 80–100 months (Figure 2). The reason for this may be that the number of AYA patients was too low after the long follow-up period (approximately 8 months later). Furthermore, as we have previously reported that the introduction of second-generation TKIs may improve treatment outcomes in high-risk patients, they may indicate that it is appropriate for physicians to choose second-generation TKIs [25]. The incidence of dose reduction in the AYA group was lower than that in the older group (Supplementary Table 1), which suggests that they were better able to tolerate the initial dose than the older group; however, the incidence of switching TKIs was higher in the AYA group (Supplementary Table 2).

We need to understand the biological and non-biological features that may be related to the clinical outcomes of AYA patients. Biological features include parameters, such as the cytokine profile, genomic variants, the immunologic profile, TKI drug levels, half-life, and other factors. Non-biological features include adherence/compliance, psychosocial issues, and other factors. Within the data for this cohort, we address the fact that AYA patients presented with a larger tumour burden at the diagnosis of CML-CP. It is possible that these findings reflect robust cytokine signalling at the diagnosis in AYA patients, and these profiles will be important to investigate in a future study. On the other hand, the ELTS score, which is recommended by European LeukemiaNet 2020 [26] as a prognostic factor, was comparable between the 2 groups. In terms of genetic variants in each group, 3 of 42 AYA patients (7.1%) and 29 of 318 older patients (9.1%) had additional chromosomal abnormalities (ACA), and we cannot conclude whether this had an impact on the outcome because of the small number of patients in the study. Regarding the immunologic profile, successful TFR has been linked to increased natural killer (NK) cells and CD8 positive T-cells, decreased regulatory T-cells, myeloid-derived suppressor cells and mature (CD86+) plasmacytoid dendritic cells **[**27–31]. It would be interesting to explore the aforementioned immunologic profile as well as cytokine profile at diagnosis in each patient group to see if they have any effect on the outcome, therefore improving the prognosis and/or leading to the acquisition of a TFR. The pharmacokinetics may differ between AYA patients and older patients and this may affect the outcome. Patients with high imatinib exposure are reported to have better CCyR, MMR and EFS rates, and more adverse events, such as fluid retention, rash, myalgia and anaemia **[**32**]**. In this study, the age of patients was reported to be weakly correlated with trough levels; however, it is considered unlikely that this has clinical significance due to the large interpatient variability in imatinib plasma trough concentrations. In another study that analysed the pharmacokinetics of imatinib and the correlation with the response and safety, age was not found to significantly affect the volume of distribution and oral clearance covariates **[**33]. Compliance is an important issue, as non-compliance has been shown to negatively affect the treatment response and outcomes [34–37]; thus, it is important to support successful treatment and to develop strategies that facilitate the simple usage of TKIs. Any non-life-threatening symptoms (e.g. fluid retention, gastrointestinal symptoms, muscle cramps, joint pain, skin rash and fatigue could be reasons for non-compliance. It is possible that AYA and middle-aged patients have poorer compliance due to socioeconomic reasons; however, this is mere speculation. On the other hand, older patients tend to have more comorbidities (e.g. cardiovascular events and malignancy), which may affect their compliance. A clinical study with a prospective cohort design is warranted to reach a conclusion regarding the relationship between adherence/compliance and the outcomes. It is also important to consider psychosocial issues, including moving due to a change of life stage, contraception, insurance or financial issues or QOL issues, which are characteristics of this particular population. One of the AYA patients died by suicide. We cannot conclude that this was related to the psychosocial character of the AYA population. A study assessing the QOL and symptom burden of patients receiving TKIs reported that there were no significant differences between the groups in terms of the effects on their daily life activities **[**38]. Combined with QOL issues, assessing the psychosocial burden of AYA patients may contribute to better management.

In conclusion, the tumour burden at the diagnosis of CML-CP is higher in AYA patients; however, the prognosis was not inferior in comparison to the older group. The MMR, EFS and OS rates of the AYA group were comparable to those in the older group. Understanding the biological and non-biological features of AYA patients with CML-CP on TKI therapy is essential for better management and may eventually improve outcomes.

## Supplementary Material

Supplemental Material

## Data Availability

The data that support the findings of this study are available from the corresponding author, TN, upon reasonable request.
