# Supplementary material for: Outcomes of adolescents and young adults with chronic-phase chronic myeloid leukaemia treated with tyrosine kinase inhibitors
Source: Ann Med. 2022 Apr 29;54(1):1244–54. doi: 10.1080/07853890.2022.2069280 (PMC9126594; doi:10.1080/07853890.2022.2069280)
Supplement: Supplemental Material [file IANN_A_2069280_SM0161.docx]

**Supplementary Table 1. Incidences and reasons for dose reduction of initial TKIs.**

|  | **Age 18-29** | **(AYA)** | **N=42** |  | **Age ≥ 30** | **N=318** |  |  |
| --- | --- | --- | --- | --- | --- | --- | --- | --- |
|  | **Imatinib** | **Dasatinib** | **Nilotinib** | **Total** | **Imatinib** | **Dasatinib** | **Nilotinib** | **Total** |
| **Total** | **N=24** | **N=13** | **N=5** | **N(%)** | **N=158** | **N=85** | **N=75** | **N(%)** |
| **Dose reduction** | **2(8.3%)** | **0(0%)** | **0(0%)** | **2(4.7%)** | **67(42.4%)** | **42(49.4%)** | **17(22.7%)** | **126(39.6%)** |
| skin rash | 1(G3) |  |  |  | 1(G1)4(G2) | 1(G2) | 1(G2) |  |
|  |  |  |  |  | 2(G3)2 |  |  |  |
| edema | 1 |  |  |  | 1(G1)1(G2)2 | 1(G1)2 |  |  |
| diarrhea |  |  |  |  | 2 |  |  |  |
| peripheral neuropathy |  |  |  |  |  | 1(G2) |  |  |
| hepatic dysfunction |  |  |  |  |  |  | 1(G1)2 |  |
| renal dysfunction |  |  |  |  | 1(G3) |  |  |  |
| pleural effusion |  |  |  |  | 1 | 1(G3)7(G2)9 | 1(G2) |  |
| fever |  |  |  |  | 1(G1) |  |  |  |
| WBC decrease |  |  |  |  | 3 | 1(G3) | 1(G3)1(G2)1 |  |
| PLT decrease |  |  |  |  | 1(G3)1(G4) | 1(G3)1(G2)1 |  |  |
| anemia |  |  |  |  | 1(G2) | 1(G2)2 |  |  |
| second malignancy |  |  |  |  | 2 | 2 | 1 |  |
| nausea |  |  |  |  | 2(G2)1(G3) |  |  |  |
| peripheral arterial disorders |  |  |  |  |  | 1 |  |  |
| purpura |  |  |  |  |  | 1(G1) |  |  |
| MI |  |  |  |  |  | 1 |  |  |
| lipase elevation |  |  |  |  |  |  | 1(G4) |  |
| amylase elevation |  |  |  |  |  |  | 1 |  |
| decubitus |  |  |  |  |  | 1(G3) |  |  |
| patient request |  |  |  |  | 6 | 1 |  |  |
| unknown |  |  |  |  | 32 | 6 | 6 |  |

The grade of each event is shown if available within the parenthesis after capital G, based on Common Terminology Criteria for Adverse Events (CTCAE) ver. 4.0.

AYA: adolescents and young adults; WBC: white blood cell count; PLT: platelet count; MI, myocardial infarction.

**Supplementary Table 2. Incidences and reasons for switching initial TKIs.**

|  | **Age 18-29 (AYA)** | | **N=42** |  | **Age ≥ 30** | **N=318** |  |  |
| --- | --- | --- | --- | --- | --- | --- | --- | --- |
|  | **Imatinib** | **Dasatinib** | **Nilotinib** | **Total** | **Imatinib** | **Dasatinib** | **Nilotinib** | **Total** |
| **Total** | **N=24** | **N=13** | **N=5** | **N(%)** | **N=158** | **N=85** | **N=75** | **N(%)** |
| **TKI switching** | **16(66.7%)** | **7(76.9%)** | **1(20%)** | **24(57.1%)** | **37(23.4%)** | **17(20%)** | **9(12%)** | **63(19.8%)** |
| resistance | 4(N)1(D) | 1(N) | 1(D) |  | 5(N)2(D) | 1(N) | 2(D) |  |
| For DMR | 5(D) |  |  |  | 3(N)2(D) | 1(N) |  |  |
| hepatic dysfunction |  |  |  |  |  | 1(G3,N) |  |  |
| drug-induced lung injury |  |  |  |  |  | 1(N) |  |  |
| muscle pain |  |  |  |  | 1(B) |  |  |  |
| renal dysfunction |  |  |  |  | 2(N) | 1(G3,IM) |  |  |
| pleural effusion |  |  |  |  | 1(B) | 1(G3,B)1(G2,B)  1(G2,N)1(N) | | |
| edema |  |  |  |  | 1(D)1(N) |  | 1(G2,IM) |  |
| nausea |  |  |  |  | 1(D) |  |  |  |
| skin rash |  |  |  |  | 1(D) | 1(G2,B) |  |  |
| diarrhea |  |  |  |  | 1(D) |  |  |  |
| cytopenia |  |  |  |  |  | 1(N) |  |  |
| dyspnea |  |  |  |  |  |  |  |  |
| drug-induced colitis |  |  |  |  |  | 1(B) |  |  |
| IgG4-related sclerosing cholangitis |  |  |  |  |  |  | 1(D) |  |
| DM |  |  |  |  |  |  | 1(D) |  |
| heart failure |  |  |  |  |  |  | 1(D) |  |
| CK elevation |  |  |  |  |  |  | 1(D) |  |
| chest pain |  |  |  |  |  |  | 1(D) |  |
| stop TKI | 1(N) | 2 |  |  | 1(D)1(N) |  |  |  |
| pregnancy | 1(IF) |  |  |  |  |  |  |  |
| patient’s request |  |  |  |  | 1(N) |  |  |  |
| unknown | 2(D)2(N) | 1(IM)2(N)1(B) |  |  | 7(D)6(N) | 1(IM)2(N)2(B) 1(IM) | |  |

The alphabets within the parenthesis are the capital letters of the TKIs or treatment that were switched to. The grade of each events is shown if is available within the parenthesis after capital G, based on Common Terminology Criteria for Adverse Events (CTCAE).

N, nilotinib; D, dasatinib; IM, imatinib; B, bostinib; IF, interferon-α; AYA; adolescents and young adults, DMR, deep molecular response; DM, diabetes mellites; CK, creatinine kinase; TKI, tyrosine kinase inhibitor.
